# Recent Advances Regarding the Therapeutic Potential of Adapalene

**DOI:** 10.3390/ph13090217

**Published:** 2020-08-28

**Authors:** Aura Rusu, Corneliu Tanase, Georgiana-Andreea Pascu, Nicoleta Todoran

**Affiliations:** 1 Pharmaceutical and Therapeutical Chemistry Department, Faculty of Pharmacy, George Emil Palade, University of Medicine, Pharmacy, Science and Technology of Târgu Mureş, 540139 Târgu Mureş, Romania; aura.rusu@umftgm.ro; 2 Pharmaceutical Botany Department, Faculty of Pharmacy, George Emil Palade University of Medicine, Pharmacy, Science and Technology of Târgu Mureş, 540139 Târgu Mureş, Romania; 3 Gedeon Richter Romania, 540139 Târgu Mureş, Romania; andreea_pascu93@yahoo.com; 4 Pharmaceutical Technology and Cosmetology, Faculty of Pharmacy, George Emil Palade University of Medicine, Pharmacy, Science and Technology of Târgu Mureş, 540139 Târgu Mureş, Romania; nicoleta.todoran@umfst.ro

**Keywords:** adapalene, dermatology, retinoids, cancer, antitumor effects, neuroprotection, antibacterial

## Abstract

Adapalene (ADP) is a representative of the third retinoids generation and successfully used in first-line acne treatment. ADP binds to retinoic acid nuclear receptors. The comedolytic, anti-inflammatory, antiproliferative, and immunomodulatory are the known ADP effects. Its safety profile is an advantage over other retinoids. ADP recently was found to be effective in the treatment of several dermatological diseases and photoaging besides the utility in the treatment of acne vulgaris. New biological effects of adapalene with therapeutic potential are highlighted in this review paper. Thus, adapalene could be a valuable therapeutic drug into the treatment of several types of cancer. Additionally, some neurodegenerative diseases could be treated with a suitable formulation for intravenous administration. The antibacterial activity against methicillin-resistant *Staphylococcus aureus* of an analogue of ADP has been proven. In different therapeutic schemes, ADP is more effective in combination with other active substances. New topical combinations with adapalene include ketoconazole (antifungal), mometasone furoate (anti-inflammatory corticosteroid), nadifloxacin (fluoroquinolone), and alfa and beta hydroxy acids. Combination with oral drugs is a new trend that enhances the properties of topical formulations with adapalene. Several studies have investigated the effects of ADP in co-administration with azithromycin, doxycycline, faropenem, isotretinoin, and valganciclovir. Innovative formulations of ADP also aim to achieve a better bioavailability, increased efficacy, and reduced side effects. In this review, we have highlighted the current studies on adapalene regarding biological effects useful in various treatment types. Adapalene has not been exploited yet to its full biological potential.

## 1. Introduction

The modern history of the retinoids begins in 1909, with the discovery of vitamin A in the egg yolk lipid extract. The retinoids group comprises vitamin A (retinol), its natural derivatives (retinaldehyde, retinyl ethers), and a large number of synthetic derivatives [1]. The first retinoids used in the treatment of acne and keratinization diseases were limited by the toxicity and adverse effects of first retinoids generation. Tretinoin was the first retinoid used topically in the treatment of acne, but with a high incidence of adverse effects. Therefore, it has been necessary to optimize these molecules by increasing administration safety. Adapalene (ADP) is a retinoid approved in 1996 by the U.S. Food and Drug Administration (FDA) for the treatment of acne (trade name Differin, producer Galderma) with fewer side effects than tretinoin [2,3].

The most common classification of retinoids is the classification in generations. The first generation includes vitamin A and some synthetic derivatives (Table 1), among the most used, are tretinoin and isotretinoin. The second generation of retinoids comprises acitretin having an aromatic cyclic moiety into the chemical structure, and third generation contain polyaromatic compounds (ADP, tazarotene) [4]. After the discovery of specific retinoid receptors, the third generation was created with various chemical structures which have optimized the selective receptors binding [5,6]. Retinoid nuclear receptors group comprises retinoic acid receptors (RAR-retinoic acid is natural ligand), and retinoid X receptors (RXR-9-cis-retinoic acid is natural ligand) [7]. Recently was approved trifarotene, a fourth-generation representative. Trifarotene is a new selective RAR-γ agonist and has 20-fold higher selectivity versus RAR-α and RAR-β receptors [8,9].

Numerous reviews have been focused on ADP and comparative studies of retinoids [1,3,5,10,11,12,13,14,15]. Due to favorable clinical observations, ADP is increasingly used *off-label* as in the treatment of lichen spinulosus [16], childhood acanthosis nigricans [17], plantar warts [18,19], alopecia areata [20], and many other conditions which will be addressed in an individual section of this review.

The known biological effects are useful to treat acne vulgaris but also other dermatological conditions. Recently, a depigmenting effect was reported in some studies regarding acanthosis nigricans treatment [17]. Additionally, a favorable impact on differentiation and maintenance of hair follicles [20], and an inhibitory action on melanogenesis was very useful in the treatment of photoaging [21]. So far, the antiproliferative effect of ADP was studied very poorly, and the mechanism of action is not very well understood. The antiproliferative effect of ADP can be exploited in the treatment of some cancer types [22,23,24,25,26]. In addition to topical combinations of ADP and other active pharmaceutical ingredients (APIs) [20,27,28], new therapeutic combinations with orally administered APIs were also reported with good effectiveness [29,30].

Nowadays, the challenge remains to design new ADP analogues with therapeutic potential [31,32]. The chemical structure of ADP may be the basis for research to develop new compounds useful in different therapeutic areas as an anticancer therapy, neuroprotection, antibacterial therapy, etc.

The literature data used in this paper were collected via Claryvate Analytics Web of Science, PubMed, and Science Direct to identify all relevant and the most recent studies regarding new biological effects of ADP and new forms that optimize ADP properties. Search terms were as follows: “adapalene” (title), “retinoids” (topic), in different combinations (topic) with “dermatology”, “cancer”, “anti-tumor effects”, “neuroprotection”, and “antibacterial” (keywords from MeSH browser), exclusive “analysis methods”. Depending on the data obtained, other combinations of specific terms were used.

The objective of this review is to highlight the new biological effects of ADP, which has not been exploited yet to its full potential. ADP has already been used *off-label* in several diseases other than acne vulgaris. The structure of this paper comprises the updated data regarding ADP in the frame of retinoid class, mechanism of action, and structure–activity relationships (SAR) of ADP, biological effects, and the potential to treat numerous diseases, adverse reactions, and toxicity. Recent studies were analyzed to highlight the enhanced therapeutic potential of ADP in different combinations with other APIs and innovative pharmaceutical formulations.

## 2. Physicochemical Properties of ADP

ADP is a stable synthetic derivative of the naphthoic acid which belongs to the class of retinoids [3,12]. Structurally, ADP contains adamantane (tricyclo [3.3.1.1] decane) and methoxyphenyl, two chemical groups that allocate particular physicochemical and biological properties. Related physicochemical properties of ADP are presented in Table 2.

ADP has the advantage of light stability, inclusive in the presence of benzoyl peroxide in the useful combinations for acne treatment [6]. ADP is more stable exposed to light and the oxidation processes than tretinoin [13]. In a stability study, it was shown that ADP is stable in 2 M NaOH solution (boiled for 2 h), and is less stable in acidic condition. Thus, in 0.3 M HCl solution after 10 min of boiling, ADP was 28% degraded. In oxidative conditions (heating at 80 °C for 10 min with 30% hydrogen peroxide solution) ADP was 30% degraded. The exposure to UV light (254 and 366 nm) degraded 25% of ADP in 12 h [6].

## 3. Mechanism of Action

The action mechanism of retinoids is based on specific binding to retinoid receptors. Retinoids that are targeting RARs affect cell differentiation and proliferation [6]. In this category, along with ADP are tretinoin and tazarotene, successfully used in the treatment of acne, psoriasis, and photoaging [6,38]. Other retinoids targeting RXRs induce apoptosis, such as alitretinoin and bexarotene, compounds that are useful in the treatment of mycosis fungoides and Kaposi sarcoma [6,38].

ADP selectively binds to RARs but does not bind to cytosolic binding proteins of retinoic acid; thus, activating genes responsible for cell differentiation. Characteristically, ADP has a high affinity for RAR-γ receptors, which are in the epidermis, and for RAR-β which are mainly in dermal fibroblasts [3], but is not very selective RAR-γ agonist as trifarotene [9]. Thereby, due to the specific binding of RARs (RAR-γ and RAR-β), ADP inhibits cell proliferation similar to tretinoin. Although the action mechanism is not fully clarified, topically applied ADP modulates keratinization, inflammation, and differentiation of follicular epithelial cells. Accordingly, the formation of microcomedones and inflammatory lesions associated with acne vulgaris is reduced [3].

In a study conducted on hamster sebocytes, experimental evidence pointed out the inhibitory action of ADP regarding sebum accumulation. This action is related to the transcriptional suppression of diacylglycerol acyltransferase 1 (the enzyme of triacylglycerol synthesis), and perilipin 1 (lipid droplet-associated protein). Additionally, ADP acts as an inhibitor to sebum storage droplet formation at the level of differentiated sebocytes by insulin, 5α-dihydrotestosterone (5α-DHT), and peroxisome proliferators activating receptors (PPARγ) [50].

## 4. Structure–Activity Relationships (SAR)

The natural retinoids present three essential structural components: (1) a p-ionone ring (a lipophilic moiety), (2) an isoprene chain susceptible to enzymatic and non-enzymatic isomerization, and (3) a polar moiety sensitive to oxidative processes [51]. SAR studies of the first two generations retinoids showed a critical role of double bonds alternatively arranged to the simple ones (e.g., tretinoin and isotretinoin). This type of structural conformation confers flexibility to molecules and offers the possibility of interacting with multiple receptors (a non-selective action). Better selectivity was assumed to be based on the hypothesis of more rigid conformational molecules [38,52] which was later obtained in the case of third-generation compounds, including ADP. Thus, ADP has four rotatable bonds and no stereoisomers comparative to tretinoin (five rotatable bonds and 16 stereoisomers) [53,54]. Structurally, ADP is a naphtoic acid derivative and consequently is more stable to light exposure, more resistant to oxidation, and has decreased irritative side effects than the first two generations of retinoids [55].

**Figure 1:**
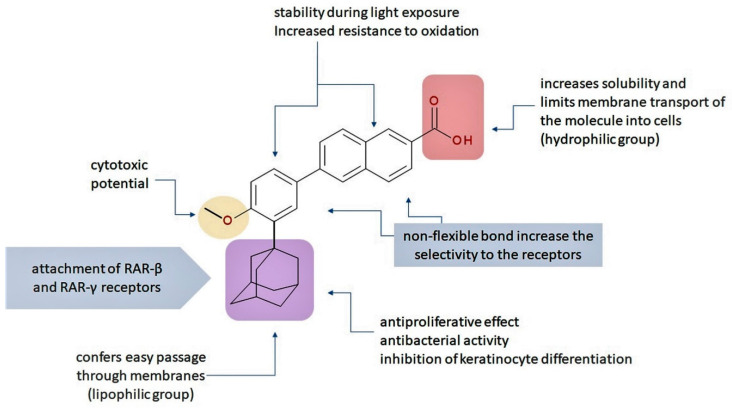
Critical aspects of structure–activity relationships on adapalene (ADP).

The valuable chemical moiety that increased the performance of the ADP molecule is adamantane nucleus. Adamantane is an essential structural component found in many other compounds, e.g., amantadine (antiviral and antiparkinsonian drug), rimantadine (antiviral drug), memantine (a drug used in Alzheimer’s disease treatment), tromantadine (antiviral drug), vildagliptin (oral anti-hyperglycemic agent), and saxagliptin (oral anti-hyperglycemic agents) [2,43]. The ADP attachment of RAR-β and RAR-γ is performed via the adamantane substituent (Figure 1) responsible for the inhibition of keratinocyte differentiation [2,38,43].

Thus, consequently to ADP and tazarotene discovery, many retinoids with specific and selective action on RAR subtype receptor have been synthesized. Some retinoid molecules are designed for the treatment of psoriasis, cancers, and mucocutaneous toxicity (Accutane-mediated). The adamantane moiety plays an essential role in the antiproliferative effect. Due to its hydrophobicity, adamantane interacts with lipid components of bacterial membranes, probably the key of an antibacterial effect [32]. The replacement of 4-methoxyphenyl with 4-hydroxyphenyl group in the structure of ADP leads to CD437, CD150 analogues. Both analogues were found to have antimicrobial activity against MRSA, and synergism in combination with gentamicin (in vivo study on mice) [56]. Additionally, CD437 presented antimicrobial activity against *Enterococcus faecalis* and inhibited biofilm formation [57]. Adarotene (ST1926) (another analogue of ADP with a 4-hydroxyphenyl group) [58] exhibited an inferior antimicrobial activity versus CD437 and CD150 analogues. Consequently, the two polar groups (carboxyl and hydroxyphenyl) of these designed analogues are essential for the antimicrobial activity [56]. Thus, CD437 and adarotene proved to have also a cytotoxic effect, which is a significant disadvantage in the development of new antimicrobial agents [56,59,60]. The 4-methoxyphenyl group may be involved in the antiproliferative effect, as suggested by a recently published review regarding SAR studies of natural and synthetic antimetastatic compounds [61]. Clarification of the mechanism of action of novel retinoids on their targets will contribute to optimizing therapies of known conditions and the discovery of new retinoids [38].

## 5. Biological Effects

ADP is approved for the acne vulgaris treatment. Acne vulgaris is a chronic, inflammatory disease of the pilosebaceous unit, characterized by comedones, papules, pustules, nodules, and scars [5,12]. There are several essential factors in the pathogenesis of this disease: higher sebum production, microbial flora changes, abnormal keratinization of skin, and inflammation. ADP is similar to tretinoin regarding efficacy, but it is more stable and lipophilic [6]. The known and potential biological effects of ADP are presented below.

### 5.1. Anti-Inflammatory and Comedolytic Effects

Pharmacological and preclinical studies of ADP have demonstrated a comedolytic and anti-inflammatory activity [31]. ADP interferes in the inflammatory process by inhibiting lipooxygenase and oxidative metabolism of arachidonic acid [10]. Some studies suggest that topical ADP therapy can achieve clinically significant improvements in treating inflammatory acne [11,62,63]. A recorded phenomenon is that the percentage of young females with acne is increasing versus adolescents with acne. A 0.3% ADP gel proved to be efficient in the treatment of young women with acne [64]. The incidences of clinically relevant improvement of inflammatory acne were with 34% higher in the ADP compared with the vehicle [11]. These studies support ADP usefulness in the treatment of acne vulgaris. In contrast, other studies conducted on rats for anti-inflammatory effects of ADP 0.1%, concluded that ADP has no statistical support for anti-inflammatory activity [60,65]. In addition, in a recent study it was demonstrated that ADP is not effective in acne-like rash associated with anti-epidermal growth factor receptor therapies [66].

### 5.2. Keratolytic Effect

Treatment of acne vulgaris with ADP or other retinoids seeks to remove these factors and reduce or eliminate acne lesions [45,67]. A 0.1% ADP is more keratolytic than benzoyl peroxide [68,69]. These effects of ADP reported efficacy in hyperkeratosis treatment. At the same time, ADP has a deficient percutaneous absorption in the corneum layer, resulting in more prolonged action in the epidermis and hair follicle, a critical therapeutic target in acne vulgaris [10]. ADP gel associated with solid lipid nanoparticles showed higher skin hydration and occlusion effect, which results in a higher accumulation of the drug in the skin [69].

### 5.3. Immunomodulatory Effect

Several studies have been reported the immunomodulatory potential of some retinoids, including ADP [31,70,71,72]. The immunomodulatory effect of ADP is based on inhibition to the leukotriene production, lipoxygenase pathways, and oxygen free radicals released from polymorphonuclear leukocytes (derived from rabbits) [71]. In addition, ADP inhibits human chemotaxis of polymorphonuclear leukocytes and the expression of mammalian toll-like receptor 2(TLR-2) on human monocytes [71,73]. This mechanism of action is additional to targeting RAR receptors.

Although, the main therapeutic intention of ADP is to treat acne vulgaris and the biologic effects of ADP have demonstrated their utility. The *off-label* uses of ADP are summarized in Table 3.

Thus, ADP was reported to be used with good results in the treatment of childhood acanthosis nigricans, epidermolytic ichthyosis, molluscum contagiosum, Darier disease, Fox–Fordyce disease, Dowling-Degos disease, pigmentary disorders, actinic keratoses, or alopecia areata, etc. [15,84]. ADP is useful in the treatment of rosacea and rosacea-like perioral dermatitis [72].

A pilot study has shown the efficacy of topical 0.1% ADP gel in the treatment of hyperpigmentation on the neck conducted in patients diagnosed with childhood acanthosis nigricans. The mean skin color ratio of the therapeutic side was significantly decreased with a skin improvement to over 60%. Treatment underwent with minimal skin irritation [17]. Another study reported treatment of epidermolytic ichthyosis with topical ADP. The results showed that ADP inhibits the proliferation of keratinocytes [82]. Thus, for pediatric patients with epidermolytic ichthyosis, ADP attenuates facial lesions and improves facial skin appearance.

Fox–Fordyce disease is an inflammatory dermatosis characterized by follicular papules, brownish skin color and is localized most often at armpits. An improvement in manifestations of Fox–Fordyce disease has been recorded when 0.1% ADP gel was topical applied [74]. The role of ADP was also shown in the treatment of pityriasis versicolor, a fungal infection of the stratum corneum [85,86]. Comparing with ketoconazole, ADP was the favorable option, but the therapeutic mechanism remains to be discussed. Additionally, topical treatment with 0.1% ADP gel has visibly improved localized lesions in patients with linear Darier’s disease [77,78,79], lichen spinulosus [16], or Dowling-Degos disease [81]. Other results revealed the efficacy and safety of 0.1% ADP gel in combination with mometasone furoate 0.1% cream for alopecia areata treatment [20]. In addition, 0.1% ADP gel is a safe [18] treatment for plantar warts and may help eliminate lesions faster than cryotherapy or other modalities available [19]. The 0.3% ADP has been recommended as an effective and safe treatment in Chilean women with cutaneous photoaging [21]. Another rare disease, trichodysplasia spinulosa was successfully treated with 0.1% ADP gel in combination with oral valganciclovir to a patient who received a kidney transplant [87].

### 5.4. Antiproliferative Effect

It is known that all retinoids have an essential role in cell growth and differentiation [88]. The ADP efficacity in the treatment of cervical intraepithelial neoplasia was demonstrated. ADP was the most effective in the treatment of level 2 cervical intraepithelial neoplasia [75].

Other studies reported that ADP could be used for the treatment of human colorectal cancer [22,89]. The in vitro effect of the ADP on human colorectal cancer cells was evaluated. ADP has an antiproliferative effect on human colorectal cancer cells and may contribute to the therapy of colorectal cancer [22]. The antiproliferative effects of ADP were examined in vivo (mice) on xenograft tumors derived from human colorectal cancer cells subcutaneously [89]. The results showed that oral administration of ADP 20 mg/kg inhibits the activity of cyclin-dependent kinase 2 in colorectal carcinoma, induces antitumor activity, and dose-dependently inhibited tumor growth [89]. The antitumoral effect of ADP, assessed by measuring DNA synthesis and apoptosis on hepatoma cells, was investigated [23]. It was shown that ADP inhibits hepatoma cell growth in vitro and induced apoptosis in the examined cell to over 79%, after 72 h incubation. In a recent study it was proved that ADP had a non-competitive inhibitory activity against glutamic-oxaloacetic transaminase 1; consequently, ADP had inhibitory activity against ovarian cancer ES-2 cells [90]. The proliferation of melanoma cells was successfully inhibited by ADP versus other retinoids as all-trans-retinoic acid, isotretinoin, acitretin, and bexarotene. The mechanism of induction of apoptosis was S phase cell cycle arrest [25]. Additionally, ADP acted inhibitory to the HaCat cells, being superior to other retinoids (all-trans-retinoic acid and isotretinoin from first-generation, acitretin from the second generation, and tazarotene and bexarotene from the third generation). The protein expression of the marker γ-H2AX, a DNA damage marker, was upregulated by ADP [26].

### 5.5. Neuroprotector Effect

The retinoid signaling is known to be essential for neurodevelopment and the normal function of the adult CNS. Some neurodegenerative diseases could be a consequence of dysregulation of retinoid signaling. In a recent study on healthy mice it was demonstrated that nanoparticles encapsulated ADP administered intravenously are bioactive in the CNS (minimum 24 h). ADP and retinoid-modulating therapies could be an alternative to the treatment of CNS diseases in the future [91].

### 5.6. Antibacterial Activity

Recently, the antibacterial activity of ADP mixed with tea tree oil loaded nano-emulsion against *Propionibacterium acnes* was investigated. The results present a significantly lower minimum inhibitory concentration (MIC) value [92]. Additionally, one analogue of ADP shows antibacterial activity against methicillin-resistant *Staphylococcus aureus* (MRSA), probably due to adamantane that intercalates into the lipidic bacterial membrane [32]. Starting from the antibacterial potential of ADP, compounds with increased efficiency can be designed in the near future, with potential in the treatment of dermatological infections. Already two ADP analogues, CD437 and CD150 (with a 4-hydroxyphenyl group instead of 4-methoxyphenyl) were found to have antimicrobial activity against MRSA, and synergism in combination with gentamicin [56]. However, the antibacterial activity of ADP has not been extensively studied.

### 5.7. Other Effects

Recently, ADP gel (non-specified concentration) successfully treated a case of 27-year-old man that suffers from acquired idiopathic partial anhidrosis. The mechanism of action is decreasing the plug at the acrosyringium, preventing ductal blockage by decreasing staining of dermcidin (in the sweat ducts), and increasing expression of cholinergic receptor muscarinic 3 (in sweat glands), and consequently ameliorating sweat delivery [93]. The increased understanding of the biological functions and mechanisms of action of ADP is likely to result in improved treatments and identification of new retinoid therapeutic targets.

## 6. Pharmacokinetic Data

ADP can be safely administered topically because their absorption into the skin is deficient. The metabolism of ADP in animals and humans is not fully elucidated. The ADP transformations on animals occur by O-demethylation, hydroxylation, and conjugation; the excretion occurs mainly biliary [2,3,43,47,94]. After topical application, ADP is concentrated in the corneum layer, most likely due to lipophilic properties. Only small amounts of ADP reach the epidermal layers and consequently, in the circulation [94]. Thus, the ADP bioavailability is limited in the skin and appendages as a consequence of high lipophilicity and p*K*_a_ value (Table 2) [45]. The modern pharmaceutical formulations of ADP presented in a separate subsection of this review are meant to overcome this disadvantage.

## 7. Side Effects, Toxicity, and Teratogenicity

In general, topical pharmaceutical forms containing ADP are well tolerated in the treatment of acne vulgaris, even for teens [95,96]. Comparative to other topical retinoids, ADP has better tolerability [5]. Thus, 0.1% ADP is less irritating than tretinoin and is better tolerated than combinations of tretinoin/isotretinoin and erythromycin [6,97]. The reported common side effects of ADP are classified as mild adverse reactions and comprise photosensitivity, redness, erythema, dryness, skin discomfort, pruritus, desquamation, and stinging/burning (Table 4) [84,96,98]. After two weeks of treatment, the intensity of side effects regularly decreases [96]. Drug formulation is related to time-control absorption and the concentration of ADP influences the severity of side effects [96]. Two similar pharmaceutical formulas have been prepared regarding tolerability and acceptance (0.1% cream and 0.1% lotion) [99]. Additionally, microsphere ADP gel was better tolerated compared to ADP gel (0.1%), keeping the same effectiveness [100]. One case of allergic contact dermatitis in treatment with ADP gel (0.1%) was reported [101].

Acute retinoid toxicity is similar to vitamin A poisoning with the following the most common signs: dry skin, conjunctivitis, reduced night vision, nosebleeds, inflammatory bowel disease flare, hair loss, musculoskeletal pain, serum lipids and transaminases alterations, pseudotumor cerebri, hypothyroidism, and mood alterations. The selective RARs retinoids are more commonly associated with mucocutaneous and musculoskeletal symptoms, while selective RXRs retinoids induce more physicochemical changes [6]. Oral retinoids are suspected of producing neuropsychiatric disorders (anxiety, depression, mood changes), but ADP is used as a topical retinoid, and its systemic absorption is negligible [102]. The oral retinoid compounds are known as teratogens. Therefore, these compounds are contraindicated in pregnancy or in women wishing to become pregnant. ADP is classified in C category risk (Food and Drug Administration-Pregnancy Categories) [6,98,102,103]. In contrast, in a recently published paper, the topical tretinoin is considered safe as an embryotoxic agent [104]; more studies are needed to clarify this essential issue.

## 8. Combinations of ADP with Other APIs

ADP has excellent stability and reduced absorption through the skin. Thus, interactions with other systemic drugs are unlikely [2,3,43]. Consequently, it can be combined with other APIs (Table 5). ADP is most commonly used in combination with benzoyl peroxide in various topical applications useful in the treatment of acne [2,105,106].

There is proof that ADP acts as a penetration enhancer if the 0.1% gel is applied 5 min before the 1% clindamycin phosphate gel [107]. In addition, ADP acts synergistically with ascorbic acid used in the treatment of acne based on increasing collagen synthesis, antioxidant and depigmenting effects. Besides, ascorbic acid is useful for decreasing common adverse reactions of monotherapy with ADP [108]. Nadifloxacin, a novel topical fluoroquinolone, proved to be a successful partner in topical combination with ADP for treatment of acne in adolescents and adults. This combination was efficient not only in acne treatment but also in decreasing ADP side effects [109,110,111].

Several treatment regimens of oral drug combinations with ADP (in topical formulations) have been published (Table 6). Many combinations are more efficient than ADP alone, the illustrative example being ADP and benzoyl peroxide [112]. A meta-analysis proves the efficacity of ADP (0.1%) in combination with benzoyl peroxide (2.5%) mainly to the treatment of moderate acne vulgaris [113]. Another example of combination with therapeutic success is topical ADP and oral azithromycin or topical ADP-benzoyl peroxide combination and oral azithromycin [114].

## 9. Analog of Retinoids

Discovery of RXR receptors brought new perspectives regarding the treatment of hyperglycemia, consequently to a study conducted on animal models of type II diabetes [38,120]. Obesity, insulin resistance, and diabetes could have an alternative to treatment with molecules as RXR agonists and antagonists (rexinoids) [121]. The (hetero)arotinoids, (hetero)aromatic retinoids are stable ligands facile to obtain [122].

## 10. The Pharmaceutical Formulation in Optimizing the Biological Properties of ADP

ADP is generally available in two formulations: gel (1%, 3%) and cream (1%) [13,95,113,123]. A recent study presented that 0.1% of ADP gel could be combined with intense pulsed light for a better efficacity in the treatment of acne [124]. The novel pharmaceuticals containing ADP are presented in Table 7.

Topical emulgels with ADP are modern pharmaceutical forms that can replace gels and creams in a friendlier manner [125]. Additionally, ADP has been loaded into an innovative microemulsion formula that proves to facilitate a transfollicular drug delivery into the skin [48]. New microemulsions containing natural alkyl polyglucosides (as “green” surfactants) were developed in order to release intradermal ADP [126]. In addition, microparticles of poly(ε-caprolactone) loaded with ADP (10% and 20%) is another recently developed formulation [127]. Poly-ε-caprolactone nanospheres containing ADP were embedded successfully in a hyaluronate gel according to the ex vivo (on human skin for retention in the epidermis and dermis), in vitro (on human dermal fibroblasts for skin irritation), and in vivo studies (on rabbits for tolerability) [128]. A nano-emulsion based on tea-three oil containing 0.1% ADP was prepared and tested in vitro, ex-vivo, and in vivo experiments. ADP dermal delivery through the skin was found to be superior compared to conventional ADP formula, and also an additional antibacterial activity was demonstrated [92,129].

Some formulation studies were focused on solid lipid nanoparticles containing ADP (0.3%–1%). These formulations presented some advantages such as greater skin hydration and occlusion effect compared to conventional gel, improved therapeutic efficacy, and reduction of side effects [69,130].

Knowing that acne-prone skin has a more acidic pH than healthy skin, ADP was successfully encapsulated in acid-responsive polymer nanocarriers and in vitro permeation study was published [131]. For delivery in the hair follicle and upper epidermis, ADP was included into a particular polymeric nanocarrier (nanospheres based on tyrosine, TyroSphere). The obtained results (in vitro and ex vivo) were auspicious in terms of delivery of hydrophobic drug and reduction of skin irritation [132]. Additionally, ADP was incorporated in polymeric micelles, based on d-α-tocopheryl polyethylene glycol succinate diblock copolymer with in vitro promising results [133].

Besides the formulations with ADP as a unique API, the most used combination in acne therapy is ADP and benzoyl peroxide (ADP 0.1% or 0.3% and benzoyl peroxide 2.5%) [45]. A liposomal gel was designed for the combination of ADP and benzoyl peroxide and superior bioavailability and decreased intensity of side effects were observed in the animal experiment comparative to free ADP, benzoyl peroxide, and Epiduo combination [134]. A recently reported pharmaceutical form comprises benzoyl peroxide nanocrystals into ADP-loaded solid lipid microparticles in the attempt to reduce the side effects of the combination of the two APIs [135,136].

Although ADP is known to be administered predominantly topically, a new formulation technique proposes encapsulation of ADP within lipid and polymer blended polyester nanoparticles to intravenous administration. This delivery system allows activation of retinoid signaling in the CNS, as proved in the experimental animal model (healthy mice) [91]. 

## 11. Conclusions

ADP is a third-generation retinoid with proven effectiveness in the treatment of acne vulgaris. The action mechanism is not fully known. The action mechanism and SAR studies suggest the biological potential that has not been fully exploited. Thus, ADP is used successfully *off-label* in the treatment of numerous dermatological conditions and photoaging. Recently, the antiproliferative effect of ADP has been demonstrated. Therefore, ADP has promising potential to be used in the treatment of some cancer types. In addition, if an appropriate intravenous formulation is used, ADP may be useful in the treatment of neurodegenerative diseases. The antibacterial activity of ADP and its analogues has been briefly explored, requiring further studies. Therapeutically, ADP is an interesting molecule that needs to be highlighted by new studies on its biological effects.

## Figures and Tables

**Table 1:** Classification of retinoids by generations.

Retinoid Generation	Administration/Indications	Representatives	Chemical Structure	Receptor(s)	References
I	Systemic and topical/acne vulgaris, photoaging, cosmetic ingredient	Retinol (vitamin A)	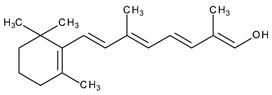	-	[33]
	Topical/acne vulgaris, photoaging	Tretinoin (all-trans-retinoic acid; vitamin A acid)	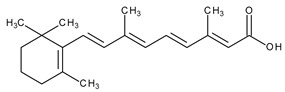	RAR-α, RAR-β, RAR-γ; RXR	[4,8]
	Systemic and topic/acne vulgaris	Isotretinoin (13-cis-retinoic acid)	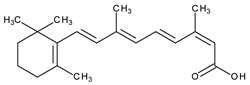	No clear receptor affinity	[4]
	Systemic and topical/Karposi sarcoma	Alitretinoin (9-cis-retinoic acid)	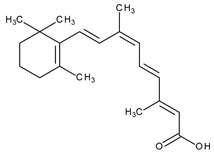	RAR-α, RAR-β, RAR-γ	[14,34,35]
II	Systemic/psoriasis	Acitretin	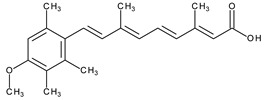	RAR-α, RAR-β, RAR-γ	[36,37]
	Topical/acne vulgaris	Motretinide	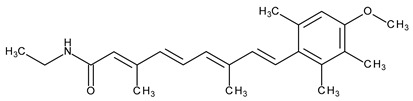	-	[4]
III	Topical/acne vulgaris, psoriasis	Tazarotene	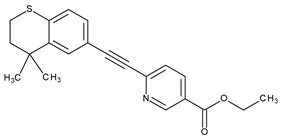	RAR-β, RAR-γ	[4,8,38,39]
	Systemic and topical/cutaneous T cell lymphomas	Bexarotene	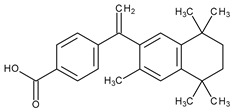	RXR	[24]
	Topical/acne vulgaris	Adapalene	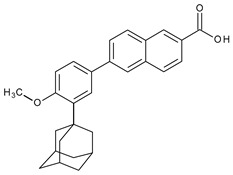	RAR-β, RAR-γ	[4,8,38]
IV	Topical/acne vulgaris (facial and truncal)	Trifarotene	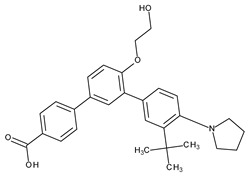	RAR-γ	[8,9,40]
	-/Photo-aging wound healing	Seletinoid G	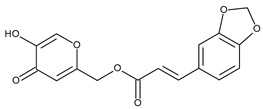	RAR-γ	[41,42]

**Table 2:** Physicochemical properties of adapalene (ADP).

Property	Description	References
IUPAC name	6-[3-(1-adamantyl)-4-methoxyphenyl]naphthalene-2-carboxylic acid	[43]
CAS number	106685-40-9	
ATC code	D10AD03 (retinoids for topical use group)	[44]
Molecular formula	C_28_H_28_O_3_	[43]
Molecular weight (MW)	412.52 g/mol	[45,46]
Appearance	White or almost white powder	
Solubility	Soluble in dimethyl sulfoxide (DMSO) (>10 mg/mL at 25 °C), dimethylformamide (DMF) (5 mg/mL at 25 °C) and tetrahydrofuran; sparingly soluble in ethanol (<1 mg/mL at 25 °C), and practically insoluble in water.	[14,46]
Melting point	319–322 °C	[14,47]
Boiling point	606.3 °C at 760 mmHg	
Density	1.2 g/cm^3^	[14]
Refractive index	1.66	
p*K*_a_	4.23; 3.99 (strongest acidic), −4.8 (strongest basic)	[14,45,47,48]
Lipophilic parameters	log P: 8.04, 8.6; 6.06, 6.47 AlogP: 6.68 XlogP: 7.7	[45,47,48]
Storage temperature	2–8 °C	[49]

**Table 3:** The *off-label* uses of adapalene (ADP).

Biologic Effect	Condition	References
Anti-inflammatory	Rosacea (reduction in inflammatory papules) Inflammatory dermatoses Fox–Fordyce disease Alopecia areata	[15,72] [20,74]
Immunomodulatory properties	Cervical intraepithelial neoplasia Actinic keratoses Actinic keratoses in solid organ transplant Pigmentary disorders Alopecia areata Plantar warts	[75] [15] [76] [20] [18,19]
Keratolytic	Acral Darier disease Milia en plaque Dowling-Degos disease Epidermolytic ichthyosis	[77,78] [79,80] [81,82] [83]
Comedolytic	Hyperkeratosis conditions	[82]
Depigmenting effect	Acanthosis nigricans	[17]
Differentiation and maintenance of hair follicles	Alopecia areata	[20]
Removal of melanin Inhibitory action on melanogenesis Potential to promote collagen synthesis	Photoaging	[21] [83]

**Table 4:** Adverse reactions during the treatment to topical gel with adapalene (ADP) (0.1%; 0.3%) [50,98,101].

Adverse Reactions	Advice for Patients
Phototoxicity	Use sunscreen products. Wear clothes that cover the treated area. Avoid exposure to sunlight or sunlamps (UV light) or minimize it.
Environmental exposure	Avoid windy or rainy weather because it may produce local irritation or skin discomfort.
Local cutaneous reactions Contact dermatitis	Avoid the use of retinoids if any lesions on the skin are present. Introduce the ADP slowly in the therapeutic routine.
Allergic/hypersensitivity reactions (face and eyelid edema, pruritus, and lip swelling)	Stop the treatment if it is necessary.

**Table 5:** The most used active pharmaceutical ingredients (APIs) in topical combinations with adapalene (ADP).

Topical Combination		Therapeutic Use	Pharmaceutical Form	Duration of Treatment	Observations	References
Content in ADP	Content in Other Active Pharmaceutical Substances (APIs)					
0.1%	2.5% Benzoyl peroxide (Normaderm^®^, Laboratoires Vichy, France - adjunctive skincare)	Mild acne	Gel for both APIs	90 days	Human patients ADP and benzoyl peroxide—in the evening Normaderm—in the morning administration	[115]
0.3%	2.5% Benzoyl peroxide	Atrophic scars in moderate or severe acne vulgaris	Gel	48 weeks	Human patients	[105]
0.3%	2.5% Benzoyl peroxide	Skin of color and mild to severe acne vulgaris	Gel	16 weeks	Human patients	[106]
1%	1% Clindamycin (phosphate)	Acne	Gel for both APIs	Pretreatment of the skin with ADP gel for 5 min	Excised rat skin Hands of human volunteers	[107]
0.1%	1% Clindamycin	Mild to moderate acne	Gel for both pharmaceuticals	4 weeks (applied gel 30 min at night)	Human patients	[116]
0.1%	2% Ketoconazole	Pityriasis versicolor	Gel (in the morning) Cream (at night)	4 weeks	Human patients	[28]
0.1%	0.1% Mometasone (furoate)	Alopecia areata	Cream (mometasone) Gel (ADP)	12 weeks	Human patients	[20]
0.1%	1% Nadifloxacin	Moderate to severe acne	Cream (nadifloxacin) Gel (0.1%)	8 weeks	Human patients ADP—in the evening Nadifloxacin—in the morning, and after ADP in the evening	[111]
0.1%	0.2% lactic acid, 0.2% glycolic acid, 0.04% citric acid, 0.01% malic acid and 0.001% salicylic acid (active day cream); 0.3% lactic acid, 0.3% glycolic acid, 0.06% citric acid, 0.015% malic acid and 0.0015% salicylic acid (active night cream)	Mild and moderate acne	Gel (ADP) Cream: active day and active night	12 weeks	Human patients ADP—three times a day in the evening	[117]

**Table 6:** Treatment regimens: oral drugs and topical pharmaceutical forms of adapalene (ADP).

Oral APIs and Doses	Topical Formulations (ADP and Other APIs Content)	Therapeutic Use	Topical Pharmaceutical Forms	Duration of Treatment	Administration	References
Azithromycin 500 mg/day	0.1% ADP and 5% benzoyl peroxide	Acne vulgaris	Gel or cream (ADP) Gel (benzoyl peroxide)	12 weeks	Azithromycin—3 days a week ADP—once daily in the morning Benzoyl peroxide—once daily in the evening	[118]
Azithromycin 500 mg/day	0.1%	Acne vulgaris	Gel	12 weeks	Azithromycin—3 consecutive days followed by 7 days rest (a 10-day cycle)	[118]
Azithromycin 500 mg/day	Erythromycin lotion (not specified%) and then ADP (not specified%)	Moderate and severe acne	Lotion (erythromycin) Not specified (ADP)	12 weeks 20 weeks	Azithromycin—3 days a week for 3 months	[119]
Doxycycline 100 mg/day	Non-specified	Acne vulgaris	Gel	12 weeks	-	[118]
Doxycycline 40 mg mg/day	0.3% ADP and 2.5% benzoyl peroxide	Severe acne	Gel	12 weeks	Doxycycline: 30 mg immediate release and 10 mg delayed release beads 25 human patients	[30]
Faropenem 600 mg/day	0.1%	Moderate and severe acne	Gel	4 weeks	-	[29]
Isotretinoin 0.5–1 mg/kg	Erythromycin lotion (not specified%) and then ADP (not specified%)	Moderate and severe acne	Lotion (erythromycin) Not specified (ADP)	12 weeks 20 weeks	Isotretinoin—5 months	[119]
Valganciclovir 450 mg (3 days per week)	0.1% ADP	Trichodysplasia spinulosa	ADP-gel	7 weeks	A 25-year-old woman (before kidney transplant)	[87]

**Table 7:** Novel topical pharmaceutical forms with adapalene (ADP) alone or in combination with other active pharmaceutical substances (APIs)

Pharmaceutical Formulation	Content in ADP	Content in Other API/APIs	Treatment of	Duration of Treatment	Observations	References
Ultrasound-mediated ADP-coated lysozyme-shelled microbubbles	13.99% ± 0.59% (in coated lysozyme-shelled microbubbles)	-	Photoaging	5 weeks	Animal model experiment (mice)	[83]
Transfersome prepared by reverse-phase evaporation	-	Ascorbic acid 15% *w*/*w*	Acne vulgaris	0, 24 h; 72 h	Animal model experiment (rats)	[108]
Solid lipid microparticle (SLM)-dispersion	0.1%	Benzoyl peroxide 2.5%	Acne vulgaris	-	Porcine ear skin experiment	[135]
Niosomal gel	95.04% ± 0.57% to 90.68% ± 0.39% (in niosomes)	-	Mild acne vulgaris	7 days	Animal model experiment (albino rats)	[137]
Liposomal formulation	97.01% ± 1.84% *w*/*w* encapsulation efficiency	-	Testing skin permeation properties	15 h	In vitro permeation studies on full-thickness pig ear skin (Franz diffusion cells)	[138]
Nanostructured lipid carriers	87.29% ± 1.6% entrapped efficiency	Ascorbyl-6-palmitate 15% *w*/*w*	Testosterone induced acne	4 weeks	Testosterone induced acne animal model experiment (Wistar rats)	[139]
Microemulsion	0.1% *w*/*v*	-	Testing penetration pathways into the skin	24 h	In vitro transfollicular delivery studies on porcine ear skin (Franz diffusion cells)	[48]
Lotion	0.1%	-	Healthy skin	3 weeks	Healthy volunteers	[99]
Microsphere gel formulation	0.1%	-	Mild to moderate acne vulgaris	12 weeks	Human patients	[100]
